# Design of a High-Speed Rotary Ultrasonic Machining Machine Tool for Machining Microstructure of Brittle Materials

**DOI:** 10.3390/mi14081544

**Published:** 2023-07-31

**Authors:** Shanhua Zhang, Manfeng Gong, Haishan Lian, Jianfeng Wu, Weijie Zhu, Zhengwei Ou

**Affiliations:** 1 School of Mechanical Engineering, Guangdong Ocean University, Zhanjiang 524088, China; 2 School of Mechanical and Electrical Engineering, Lingnan Normal University, Zhanjiang 524048, China

**Keywords:** rotary ultrasonic machining, high speed, hard and brittle materials, machine tool design, experimental study

## Abstract

Aiming at the problems of low machining accuracy and more serious tool wear in the traditional diamond grinding machining (DGM) microstructure of hard and brittle materials, this paper proposes high-speed rotary ultrasonic machining (HRUM) technology and develops a HRUM machine tool. The hardware part of the machine tool mainly includes the spindle module, micro-motion system module, ultrasonic machining tank module, and data acquisition (DAQ) system module. The LabView-based controlled machining control system, including motion selection, initialization, coarse tool setting, constant force tool setting, control machining, and coordinate display module, is developed. Comparative experimental research of the HRUM and DGM of small holes in Al_2_O_3_ ceramics is carried out in the developed HRUM machine tool. The results demonstrate that HRUM effectively reduces axial cutting forces, reduces binder adhesion, and suppresses slippage while improving tool-cutting ability and extending tool life compared to DGM under the same machining parameters. This technology has essential research significance for the high-precision and efficient machining of microstructures in hard and brittle materials.

## 1. Introduction

Hard and brittle materials such as ceramics, sapphire, and glass have a wide range of application prospects due to their superior chemical, mechanical, and physical properties. However, their inherent hard and brittle nature quickly results in defects such as chipping, cracking, and severe tool wear during processing, significantly limiting their applications. The initial ultrasonic processing technology used a high-frequency vibrating tool head to impact abrasive particles in suspension to achieve material removal [1]. Rotary ultrasonic machining (RUM) improved from conventional ultrasonic machining, which combines the material removal methods of conventional ultrasonic machining and diamond grinding machining (DGM). It has the advantages of good machining quality, higher machining efficiency, accuracy, and longer tool life, which is considered an effective machining method for processing hard and brittle materials [2,3].

The rotary ultrasonic system mainly comprises the machine body, ultrasonic power supply, and rotary vibrating tool holder. Among them, the electric energy transmission device is in development, while the ultrasonic power supply design and the ultrasonic transducer manufacturing have been relatively mature. Generally, the electric energy transmission device used for applying ultrasonic vibration is divided into two types: slip ring brush contact type and non-contact type based on the principle of wireless power transmission (WPT). In terms of contact rotary ultrasonic processing technology, in 1964, P. Legge [4] first proposed a rotary ultrasonic processing method using sintered and electroplated diamond tools and then modified a conventional machine tool by using a slip ring and brush assembly to transmit electrical energy and an autotransformer to control the motor speed in the range of 100–1800 r/min. Drilling tests on glass and ceramic workpieces concluded that RUM could drill more profound and precise holes than conventional ultrasonic machining. In 1986, Fan et al. [5] from the Eleventh Research Institute of the Ministry of Electrical and Mechanical Affairs of China developed an experimental prototype of T3030-3/ZV RUM for processing YGA laser crystal rods, with a rotating speed that can reach 1000–3000 r/min. In 2009, Zheng et al. [6] used a brush-ring-type transducer to develop the RUM machine tool, which has a speed range of 0–3500 rpm and is suitable for hole and face processing below 60 mm in diameter. In 2014, Professor Lin’s team [7] from Tianjin University successfully developed a rotary ultrasonic spindle head based on high-speed slip ring technology, which reaches a speed of 4000–6000 r/min. In 2022, Zhou [8] from the North University of China designed a parallel wheel rotating ultrasonic grinding tool system using an over-hole conductive slip ring (maximum speed 5000 rpm) and tested its performance. In 2023, Prof. Geng’s team [9,10,11] at Beihang University developed their own ultrasonic-assisted system, which transmits electrical signals through a slip ring to a piezoelectric ultrasonic transducer, a core device mounted on a BT50 tool holder, to convert electrical signals into mechanical energy. And they studied a series of machining processes such as ultrasonic-vibration-assisted milling (UVM) and rotary ultrasonic elliptical milling (RUEM).

With the advent of high-speed spindles, mechanical contacts such as slip rings and brushes are prone to wear, a short life, and the loss of electrical signal, while the high friction between them leads to vibration and limits the spindle speed. Thus, many researchers have intensively investigated contactless power transfer (CPT)/wireless power transfer (WPT) devices. The loosely coupled transformer (LCT) is widely used as a WPT system for RUM equipment due to the advantages of no wear and tear and suitability for high rotational speed [12,13,14]. In RUM, inductive power transfer (IPT) requires a small gap (d = 0.1–1 mm) to achieve a transmission efficiency of more than 90% [15,16]. Professor Guo’s team at the Guangdong University of Technology [17] independently developed five-axis rotary ultrasonic processing machine tools with a CPT system and a spindle speed of up to 0–8000 r/min. In addition, Tianjin Polytechnic University, Dalian University of Technology, and Zhejiang University have studied the WPT of the RUM system.

In recent years, high-speed ultrasonic vibration cutting (HUVC) and ultrasonic-assisted high-speed drilling (UAHD) [18,19] have successfully been applied to the high-speed machining of titanium alloys as novel machining processes. By changing the direction of tool vibration, the limitation of cutting speed is overcome, and a high cutting speed of 200–400 m/min is achieved, which results in higher cutting efficiency, improved surface quality, longer tool life, etc. [20,21,22]. The development of RUM machine tools also gradually tends to high speed, multiple axes, and systematization, improving the machining accuracy and efficiency and overcoming the problems of excessive cutting forces, severe tool wear, and the surface loss of workpiece materials triggered by traditional machining. Much of the research has confirmed that spindle speed is one of the critical factors affecting the accuracy of the RUM of hard and brittle materials [23,24,25,26,27,28,29]. If the spindle speed can effectively increase, it will improve RUM’s processing quality and efficiency for hard and brittle material microstructure. Therefore, research to further increase the spindle speed of RUM can achieve efficient, precise, low-damage, and low-cost machining of hard and brittle materials.

**Figure 1:**
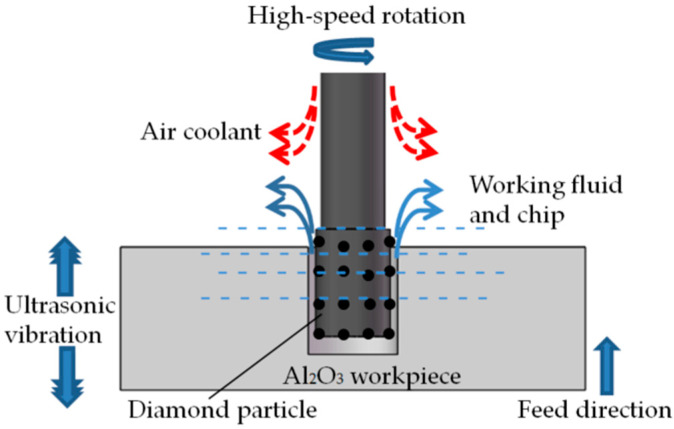
Illustration of HRUM.

Researchers are increasingly concerned about the stability of the transducer [30,31,32]. In RUM, high rotational speed may adversely affect the stability of the transducer. However, by transferring the ultrasonic vibrations to the workpiece, it is possible to increase the spindle speed while ensuring the stability of the transducer. Consequently, this paper proposes a new process of high-speed rotary ultrasonic machining (HRUM) for the microstructure of hard and brittle materials. The workpiece size in microstructure machining is generally small, allowing for ultrasonic vibration to directly act on the workpiece. To facilitate HRUM, a HRUM machine tool (spindle speed ≥ 8000 r/min) was developed. As shown in Figure 1, in HRUM, the sintered diamond tool rotates at a high speed driven by an electric spindle, while the Al_2_O_3_ workpiece is in longitudinal ultrasonic frequency vibration. The material removal mechanism is a combination of ultrasonic processing and diamond grinding removal mechanisms, incorporating hammering, abrasion, and polishing effects. Implementing ultrasonic vibration on the workpiece when machining the microstructure of hard and brittle materials can significantly increase the spindle speed, effectively reducing the machining cutting force and improving the material removal rate and machining accuracy.

## 2. The Design of the HRUM Machine Tool

### 2.1. The Overall Structure of the Machine Tool

**Figure 2:**
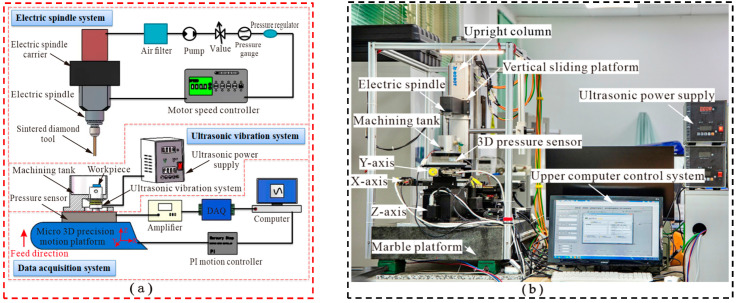
The HRUM experimental setup: (**a**) schematic diagram; (**b**) physical diagram.

The primary motion of HRUM is divided into the rotational motion of the spindle and the tool trajectory motion corresponding to the specific machining object. The machine is of C-type construction which consists of an electric spindle system, an ultrasonic vibration system, and a DAQ system, as shown in Figure 2. The electric spindle system mainly comprises a spindle carrier, an air filter, and a motor speed controller. The air filter has the purpose of cooling and purging the motor spindle. In order for the spindle to rotate, it is necessary to supply regulated air to the control unit and set the air pressure of 0.2–0.3 Mpa. The electric spindle is mounted on a vertical sliding platform through the electric spindle carrier, fixed on an upright marble column. The spindle can be moved up and down along the sliding table, driven by a servo motor. The electric spindle and auxiliary parts have a certain weight, the vertical sliding stage has a screw + double guide structure, and the selected servo motor has a self-locking function. The sintered diamond tool is locked by the electric spindle through a clamp, and the spindle drives the tool to rotate at high speed.

The ultrasonic vibration system includes an ultrasonic power supply and a machining tank. The ultrasonic power output can be adjusted by controlling the power supply. The workpiece is subject to ultrasonic vibration through the ultrasonic vibration system installed in the machining tank, which generates high-frequency mechanical vibration during the machining process. Since the vertical slide table is unable to meet the requirements of HRUM for machining microstructures with high motion accuracy of the stage, the trajectory motion to be realized by the tool during machining is controlled by the micro three-dimensional (3D) motion platform. The machining tank is mounted on a micro 3D precision motion stage via a 3D pressure transducer to meet the control system hardware requirements for the microstructure machining of hard and brittle materials. The DAQ system plays a key role in measuring cutting forces and displacements during machining. It consists of a 3D pressure sensor, an amplifier, a data acquisition card, and a micro 3D motion stage. Detailed information for the DAQ card will be provided in Section 2.2.

### 2.2. Design for Major Machine Components

**Figure 3:**
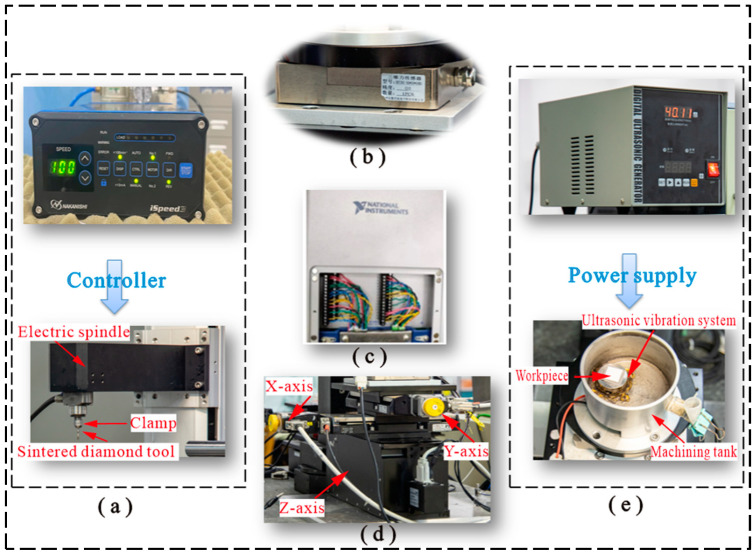
Design of key components: (**a**) NSK electric spindle system; (**b**) 3D pressure sensor; (**c**) NI DAQ card; (**d**) micro 3D motion platform; (**e**) ultrasonic vibration system workbench: machining tank and ultrasonic power supply.

The design of critical components includes the electric spindle, 3D pressure sensor, DAQ card, micro 3D motion platform, and ultrasonic vibration system, as shown in Figure 3. The electric spindle (BM320F, NSK, Tokyo, Japan) enables continuous adjustment from 1000 to 80,000 r/min and provides the power source for rotary machining, as shown in Figure 3a. The sintered diamond tool is mounted on the electric spindle through the clamp, and the tool rotates at high speed while being driven by the electric spindle.

Figure 3b shows the 3D pressure sensor (SBT301, Guangzhou Youshineng Electronic Technology Co., Ltd., Guangzhou, China), with a range of 500 N in all three axes and a combined error of ≤±1%. It is applied to constant force tool setting and machining force measurement in machining.

In any closed-loop motion control system, there is invariably a parameter as the feedback signal to achieve motion control. During the experimental motion control process, the feedback signal generally adopted as the control parameter is mainly force, speed, etc. In the HRUM process, the force signal of real-time processing is generally used as the feedback signal of the motion control system. The machining signals are acquired by a DAQ card (NI6361, NI, Austin, TX, USA), as shown in Figure 3c. The technical parameters of the acquisition card can be found in Table 1.

The hardware design of the HRUM microstructure for hard and brittle materials is based on the combination of macro displacement motion components and a micro displacement motion system. Above all, coarse tool setting is performed to make the tool move within the range of motion of the Z-axis of the micro 3D motion stage, while the micro 3D motion stage realizes the precise tool setting and machining motion. The micro 3D motion platform is shown in Figure 3d; it is fixed on a marble platform with the travels of the X-axis, Y-axis, and Z-axis of 102 mm, 102 mm, and 25 mm, respectively, and the minimum resolution of each axis is 0.1 μm.

Figure 3e shows the ultrasonic vibration system’s physical diagram. In the HRUM of workpiece vibration, the workpiece is held on the ultrasonic vibration workbench through a double-sided adhesive, which makes the workpiece achieve high-frequency mechanical vibration under the drive of the ultrasonic power supply.

#### Ultrasonic Vibration System Design

**Figure 4:**
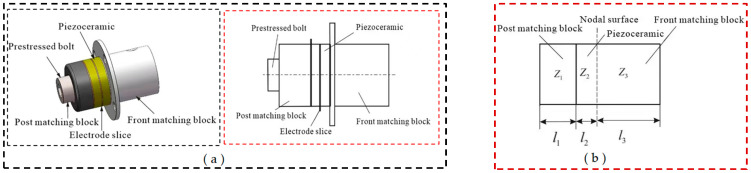
The design of microfine ultrasonic vibration system: (**a**) schematic diagram; (**b**) equivalent diagram.

The required amplitude value is minimal in microfine ultrasonic or ultrasonic-assisted processing. Generally, the microfine amplitude generated by the Piezoceramic is enough to meet the processing requirements, and there is no required amplification of the amplitude by the variable amplitude rod. The microfine ultrasonic vibration system includes the front matching block, Piezoceramic, electrode slice, post matching block, and prestressed bolt, as shown in Figure 4a. When the theoretical design is carried out, ignoring the prestressed bolt and the electrode slice, the equivalent design of the microfine ultrasonic vibration system is shown in Figure 4b. The whole vibration system is λ/2, and the nodal surface is designed at the contact surface of the front matching block and the Piezoceramic. For the back part of the nodal surface, the frequency equation is as follows:

(1) $$\tan k_{1}l_{1}\tan k_{2}l_{2}=\frac{Z_{2}}{Z_{1}}$$

According to the corresponding part of the transducer in Figure 4b, where *Z*_1_ is the wave impedance of the front matching block, $Z_{1}=\rho_{1}c_{1}S_{1}.$ *Z*_2_ is the wave impedance of the Piezoelectric$,Z_{2}=\rho_{2}c_{2}S_{2}$. *k*_1_ is the wave number of the front matching block$,k_{1}=\omega /c_{1}.$ *k*_2_ is the wave number of the Piezoelectric $,k_{2}=\omega /c_{2}$. *l*_1_ is the length of the front matching block, and *l*_2_ is the Piezoelectric. The length of the front matching block part is as follows:

(2) $$l_{3}=\lambda_{3}/4$$

The material parameters of the main parts within the microfine ultrasonic vibration system are demonstrated in Table 2, where the longitudinal wave velocity is the theoretical value. The outer diameter of the Piezoceramic,$\;D_{2}=30\;\mathrm{mm}$, the transducer is made of two piezoceramics, the thickness of the piezoceramic $l_{21}=l_{22}=5\;\mathrm{mm}$, and the vibration frequency f=40 KHz. From Equations (1) and (2), it is obtained that $l_{1}=20.1\;\mathrm{mm}$,$\;l_{2}=10\;\mathrm{mm}$, and $l_{3}=32.4\;\mathrm{mm}$. Take$\;l_{1}=20\;\mathrm{mm}$,$\;l_{2}=10\;\mathrm{mm}$, $l_{3}=32\;\mathrm{mm}$.

## 3. Machine Tool Control System Design

**Figure 5:**
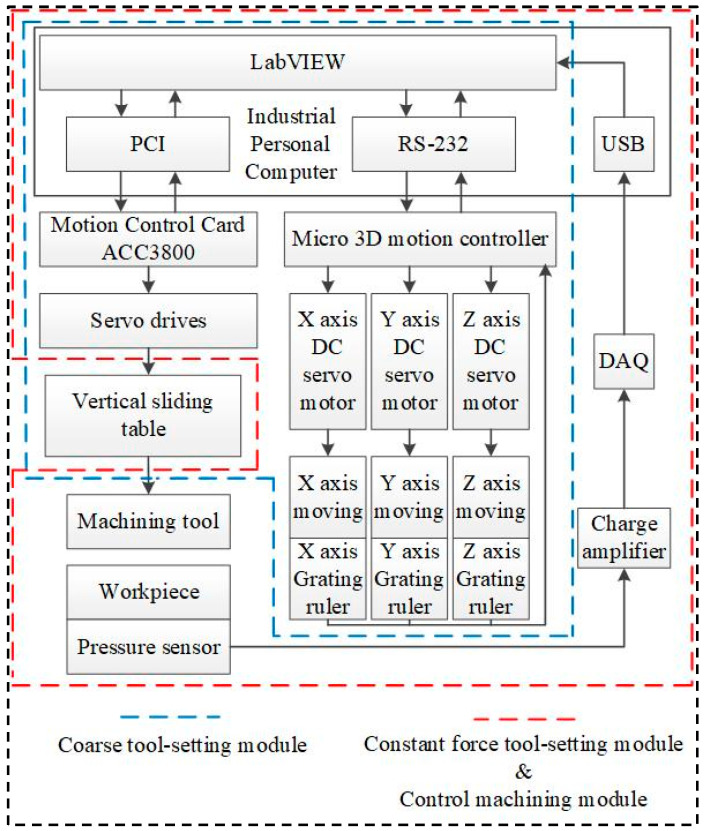
HRUM control flow diagram.

The purpose of the software design is to control the movement of each moving part of the HRUM machine according to the specific machining trajectory to complete the machining task. The concrete movements of HRUM include the vertical slide movement, the rotation of the spindle, and the movement of the micro 3D stage. To improve the reliability and fast response of the machining system, the machine adopts the two-stage control method of PC IPC + multi-axis motion control card. The multi-axis motion control card (ACC3800, Leadshine Technology Co., Ltd., Shenzhen, China) is employed to control the spindle’s vertical slide and rotational motion. The micro 3D motion platform has a motion controller connected to the PC industrial controller via RS-232. The overall control flow of the machine is shown in Figure 5.

**Figure 6:**
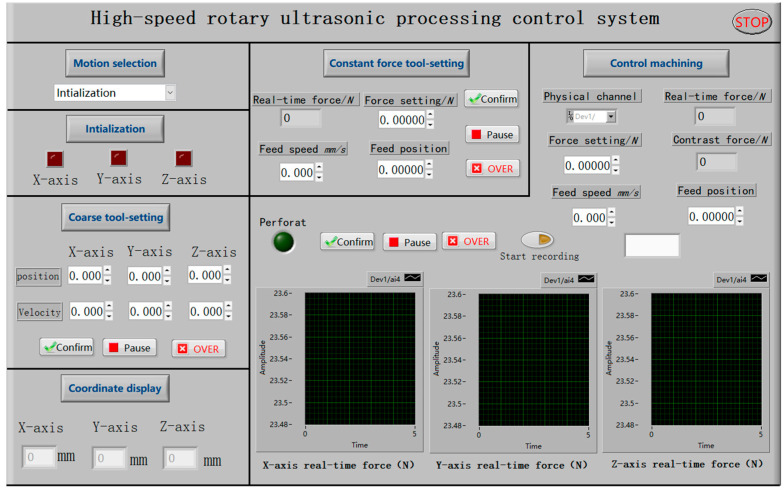
HRUM program control interface diagram.

The micro 3D motion platform plays a crucial role in ensuring the control accuracy of the motion system in the machining process. Consequently, the control program is mainly written based on the library functions of the platform. As shown in Figure 6, the program is divided into six functional modules, including motion selection, initialization, coarse tool setting, constant force tool setting, control machining, and the coordinate display module. In the specific implementation, the initialization module is responsible for the power-on and reset operation for each axis. After executing this module, different motion modules can be chosen through the drop-down menu of the motion control selection module. When one motion module is selected, the control buttons for the other modules become inactive. However, due to the limited travel (25 mm) of the Z-axis in the micro 3D motion platform, it is still necessary to control the lift of the spindle with the macro motion of the vertical sliding platform. This ensures proper tool electrode clamping and prevents damage to the machine tool.

After initialization, all axes of the micro 3D motion platform are at the mechanical origin of the platform. The machining workpiece mounted on the micro 3D motion platform is far from the tool electrode mounted on the spindle. Controlling the X-axis and Y-axis movements of the micro 3D motion platform is necessary. Additionally, the vertical sliding platform should be utilized to bring the tool and the workpiece in close proximity. The above processes are collectively called the HRUM coarse tool-setting control module. The coordinate display module provides real-time feedback on the current machining coordinates of each axis.

### 3.1. Constant Force Tool-Setting Module

The function of the constant force tool-setting module is to ensure that the tool and the workpiece have a specific contact pressure. This is achieved through the adjustment of the contact pressure. The Z-axis of the micro 3D motion stage is controlled to move upward to make the workpiece approach the tool. At the same time, the pressure sensor detects the real-time contact force between the tool and the workpiece. This force is converted into a voltage signal by the pressure sensor when it acts on the pressure sensor. The voltage signal is then amplified by the charge amplifier and input to the DQA card. If the real-time pressure value is less than the pressure setting value, the LabView program continues to control the Z-axis of the micro 3D motion platform to move upward and enter the next sampling cycle. Conversely, if the real-time pressure value is greater than or equal to the pressure setting value, the LabView program immediately brakes the Z-axis of the micro 3D motion platform.

During the sampling period, the LabView program calls the DAQ module to process the voltage signals. All the voltage values in a sampling period are calculated according to Equation (3) to determine the average voltage value. The average voltage value is then multiplied by a specific scale factor to convert it into the real-time pressure value.

(3) $$\mu =\frac{1}{n}\overset{n}{\underset{i=1}{\sum }}x_{i}$$

where μ is the average value of the voltage during the sampling period, i is the ith data point, $x_{i}$ is the voltage value of the ith data point, and n is the total number of data points during the sampling period.

### 3.2. Control Machining Module

The constant force tool-setting module is implemented prior to the control machining module, which is responsible for controlling the required motion of HRUM. Simultaneously, it records the changes in force and the coordinates of the related motion axis during the machining process, enabling subsequent data processing and analysis. Using the LabView program, the Z-axis of the micro 3D platform is controlled to perform constant speed feeding movement. The program compares the real-time pressure value collected by the DAQ module with the current maximum pressure value and the upper threshold for pressure setting. If the real-time pressure value exceeds the upper pressure threshold, the program initiates a fast retreat movement of the Z-axis, followed by a check to determine whether the axis has reached the target position. Since the cutting force decreases significantly during hole punching, if the difference between the actual time pressure value and the current maximum pressure value is larger than 5 N, it indicates that hole punching has occurred, and the Z-axis retreats immediately. At this point, the perforation signal is activated, the Z-axis stops moving, and the process is complete.

## 4. Experimental Research

### 4.1. Experimental Setup

**Figure 7:**
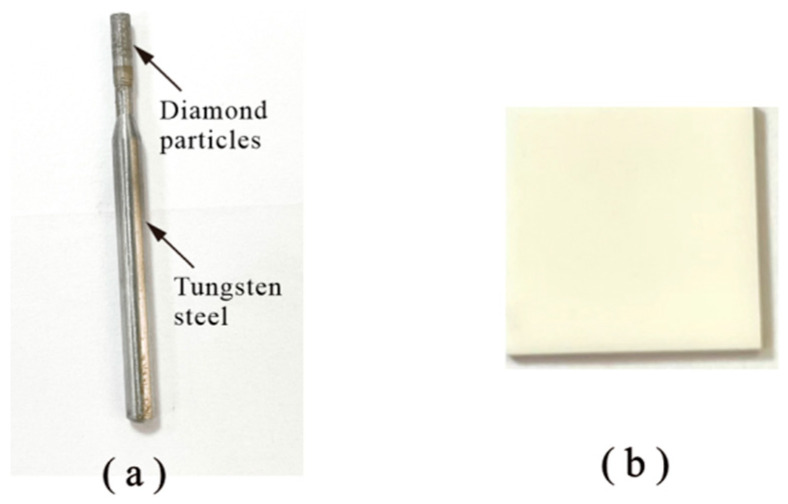
Experimental materials: (**a**) sintered diamond tool; (**b**) Al_2_O_3_ ceramic pieces.

The experiments of HRUM and DGM small holes were conducted using the HRUM machine tool. In this experiment, a 2 mm diameter sintered diamond tool (Zhengzhou Zhongtuo Abrasives Co., Ltd., Zhengzhou, China) was utilized for processing Al_2_O_3_ ceramic material, as shown in Figure 7. The ceramic workpiece had dimensions of 15 mm × 15 mm × 1 mm, with the properties listed in Table 3. To enable machining, the Al_2_O_3_ ceramic was attached to the microfine ultrasonic vibration workbench of the machining tank and submerged in the working fluid using a robust double-sided adhesive.

The process parameters were as follows: spindle speed of 10,000 r/min, feed rate 0.01 mm/s, workpiece vibration ultrasonic power output of 60%, and resonant frequency of 40 KHz. The amplitude of the output of this vibration system is mainly controlled by the output power, which is in the range of 1.0–2.8 μm. Other processing parameters are listed in Table 4. The small hole inlet chipping was observed by scanning electron microscopy, while the machining process was compared and analyzed for tool-cutting force changes.

### 4.2. Results and Discussion

#### 4.2.1. Effect of Tool Wear on Cutting Force and the Number of Holes Made

The Al_2_O_3_ ceramic small hole experiments were implemented under the same process parameters, comparing three abrasive diameter tools with and without applied ultrasonic machining conditions to research the effect of tool wear on cutting force and the number of holes made. The cutting force was detected by the 3D pressure sensor and acquired by the NI DAQ card. The sampling frequency of the acquisition card is 10,000.1 Hz, and the sampling time is 1000 s, as detailed in Section 2.2. Considering that there is no significant difference in the X/Y axial force, the experiment only investigates the variation in the Z-axis cutting force.

**Figure 8:**
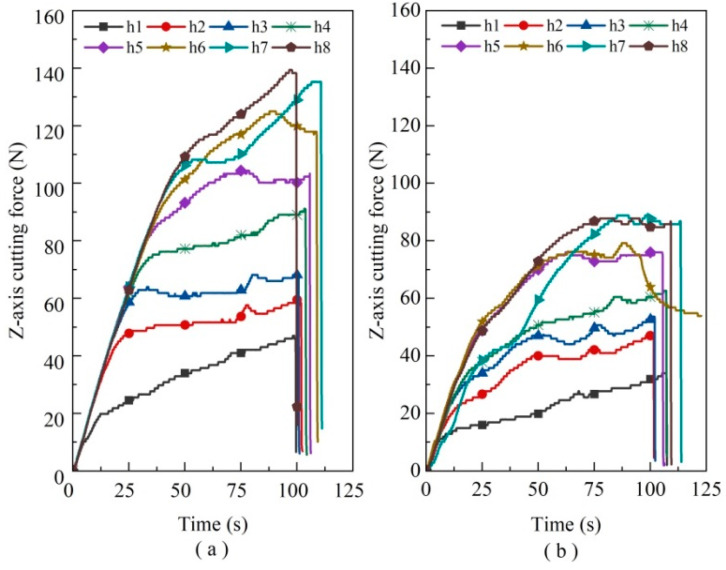
Abrasive grain 80 mesh tool Z-axis cutting force situation: (**a**) DGM and (**b**) HRUM.

When the cutting force reaches its maximum limit value, it fails to complete the workpiece piercing machining, thus indicating tool failure. For instance, the cutting force variation throughout the machining process of the 80 mesh sintered diamond tool can be observed in Figure 8 (where “h” is the abbreviation for “hole”, and the number stands for the ordinal number of holes to be machined). It is evident from the slope of the curve that the growth rate of cutting force in DGM follows a linear trend in relative time. This growth rate is significantly faster before 50 s and becomes more moderate thereafter. In contrast, the slope of the curve for HRUM gradually becomes more moderate due to its pulsed intermittent cutting nature. By eliminating chips in time, HRUM reduces cutting resistance and effectively lowers the cutting force.

**Figure 9:**
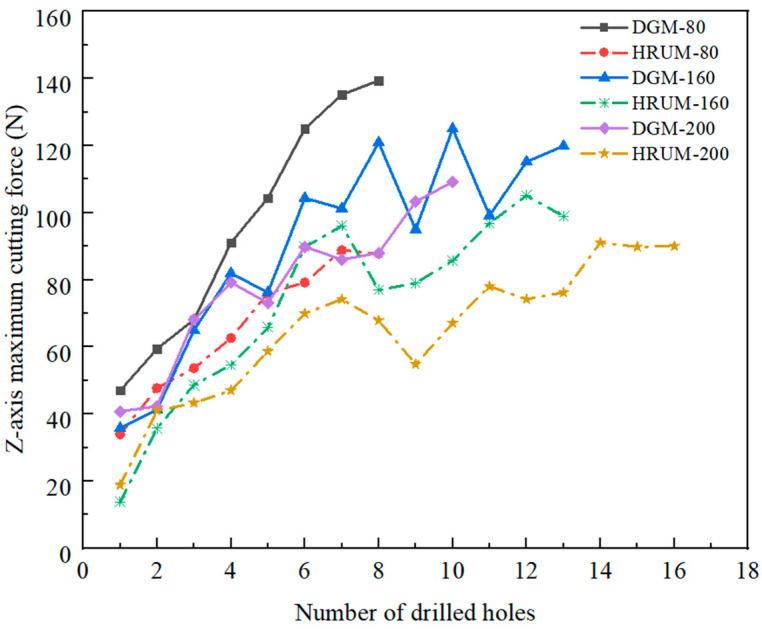
Maximum cutting force versus number of drilled holes in DGM and HRUM.

Considering the little difference in the trend of the abrasive grain diameter machining curve, the maximum machining force required for each hole machining in each group of experiments is taken into account for comparison. The effect of tool wear on the number of holes and cutting force is investigated for different grit diameters and with or without applied ultrasonic vibration, as shown in Figure 9. The experiments demonstrate that the 80 mesh abrasive grain can machine eight holes, while the 160 mesh tool can machine 13 holes. There is no difference in the number of holes machined with or without the application of ultrasonic vibration. However, compared with HRUM, DGM requires more cutting force for machining.

The tool with 200 grits demonstrates a significant difference in the number of small holes machined. The tool can machine 16 holes with ultrasound, while DGM only machined ten holes. This is primarily due to the application of ultrasonic vibration, which facilitates periodic cutting-separation contact cutting between the tool and the workpiece. This contact cutting reduces the cutting force and slows down tool wear, resulting in an increased number of holes made. Furthermore, the abrasive grain size mainly influences the cutting force. The larger grain sizes have fewer sharp edges and larger angles of the edges, which increases the tip of the abrasive grain abrasion and tool wear. Consequently, the required cutting force becomes more extensive.

The maximum cutting force required for tool processing gradually increases with the increase in small holes. After the 7th–13th holes, cutting force growth is in the fluctuation range. Diamond grain exposure height is higher in the initial processing stage. However, as the diamond grains wear rapidly, the tip area of diamond grains increases, and the exposed height becomes relatively consistent, marking the entry into the stable processing stage. As the number of small holes increases, severe wear of many abrasive grains begins to appear. Simultaneously, the tool gradually fails due to the influence of the greater bond strength of the tool binder.

#### 4.2.2. Effect of Tool Wear on the Surface Quality of the Hole

**Figure 10:**
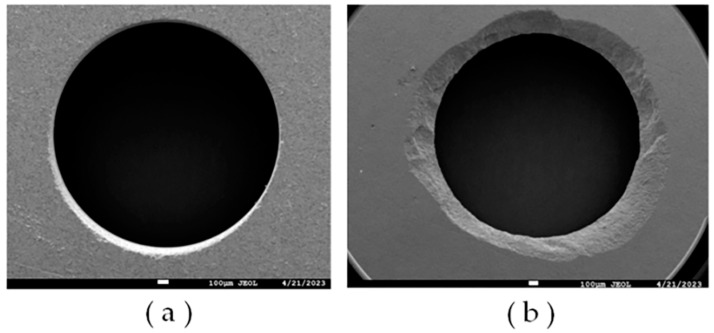
Quintessential hole-making defects in hard and brittle ceramic material. (**a**) Entrance defect; (**b**) exit defect.

Figure 10 illustrates the quintessential hole-making defects of hard and brittle ceramic materials, including the defect problems such as hole exit and entrance chipping. It is noticeable that the hole exit defects are more severe in comparison. As the number of holes made increases, the tearing defects become more severe than burrs and chipping edges. Material removal by ultrasonic vibration is nearly related to the sharpness of diamond abrasive grains. Consequently, the macroscopic force action of all diamond abrasives causes hole exit defects. During the machining process, diamond abrasive grains will wear due to friction, increasing the tip area, which causes the cutting force to increase. Meanwhile, the tool binder strength also affects the cutting force, which increases the exit chipping area. Moreover, hole exit tearing defects are generated with the strength of the axial cutting force, with the number of holes made to increase the cutting force also increasing, cutting the underlying material lost support and leading to damage at the removal of block form.

The material removal process of a single diamond abrasive typically leads to hole entrance defects, such as burrs, edge chipping, and other common defects. The hole entrance conditions after each group of experimental machining were compared and observed using scanning electron microscopy (SEM) (XL-30, FEI, Hillsboro, ON, USA) to investigate the effects of abrasive grain diameter and ultrasonic vibration on the entrance quality.

**Figure 11:**
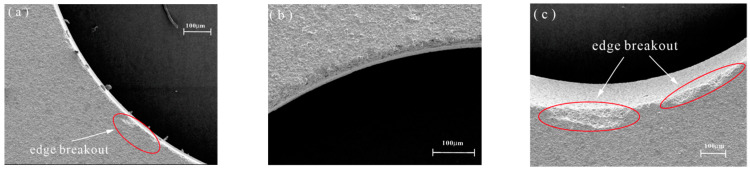

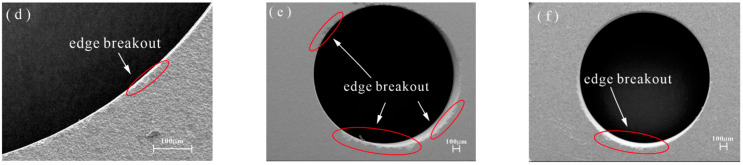
SEM images of the entrance surface at three abrasive particles in DGM and HRUM for the last hole: (**a**) DGM and (**b**) HRUM 80 mesh for the 7th hole; (**c**) DGM and (**d**) HRUM 160 mesh for the 13th hole; (**e**) DGM and (**f**) HRUM 200 mesh for the 10th hole.

The initial machining stage shows more severe edge chipping in DGM compared to HRUM, indicating that HRUM has improved in reducing edge defects. However, when examining the entrance situation of each abrasive grain tool during the machining of the last hole, as shown in Figure 11, it can be observed that the improvement effect of HRUM is not evident in the subsequent machining stages, and the same chipping phenomenon occurs. This can be attributed to the continuous wear of diamond particles on the tool during subsequent machining, leading to the occurrence of chipping. Additionally, this continuous wear also results in a significant increase in the required cutting force, which in turn affects the ultrasonic vibration.

**Figure 12:**
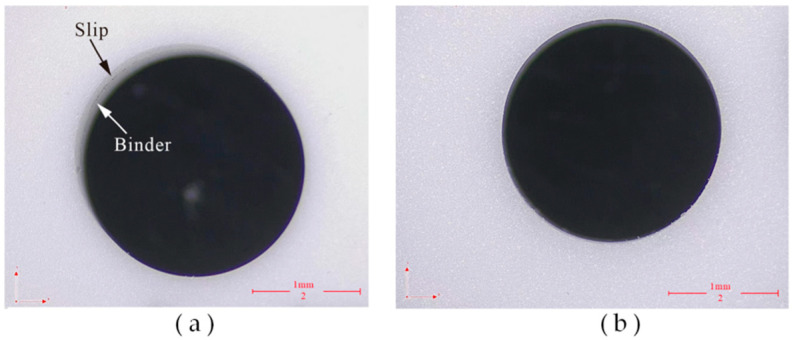
Micrograph images of the entrance surface: (**a**) DGM and (**b**) HRUM.

The overall machined surface morphology of the hole is observed through an optical microscope (CSW-H2KACL, COOSWAY, Shenzhen, China), as shown in Figure 12. It is observed that the binder is easily attached to the side of the subsequent holes in DGM. This is due to the fact that after the side diamond tip wears out, the side binder rubs against the side of the workpiece. As a result, the binder comes off in chips and adheres to the side of the workpiece. The impact effect of ultrasonic vibration helps to discharge the debris, which can avoid the adhesion of the bond to a certain extent. Additionally, DGM is more likely to slip and form a non-circular entrance in the subsequent hole processing. This occurs because it is formidable always to ensure that the tangent surface of the workpiece at the entry point is perpendicular to the spindle rotation axis in micro drilling, due to various errors. Consequently, when penetration starts, the tool easily slips on the workpiece surface, leading to hole position errors. Furthermore, the large amplitude ultrasonic vibration is beneficial in improving the cutting ability of the tool and suppressing the slipping phenomenon [33].

#### 4.2.3. Sintered Diamond Tool End-Face Wear

**Figure 13:**
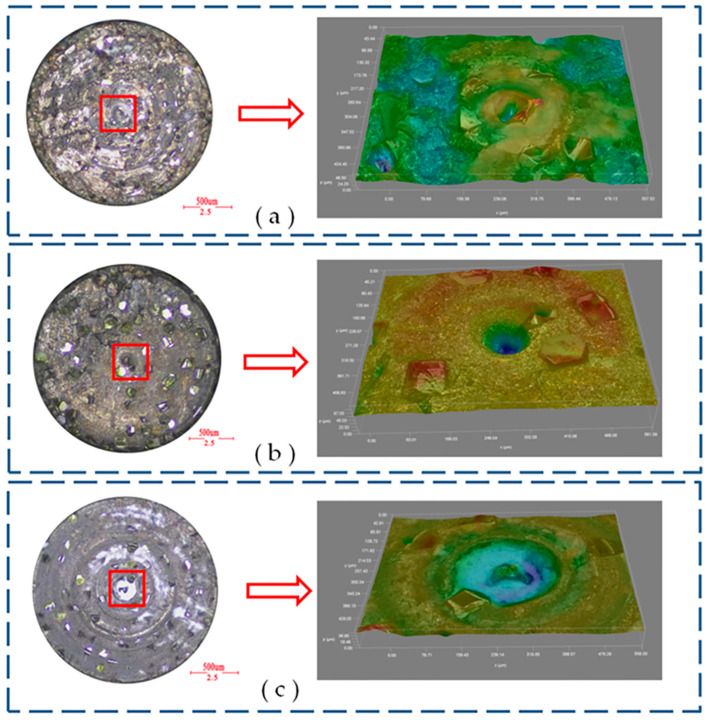
Micrograph images of the tool end face: (**a**) DGM and (**b**) HRUM for 160 mesh abrasive at the last hole; (**c**) DGM 200 mesh abrasive at the last hole.

The primary forms of abrasive failure include wear, crushing, and shedding. The cutting force on a single diamond grain is less than the bonding strength of the binder, causing continuous wear and the tip area to become more prominent. When the cutting force exceeds the bonding strength, the abrasive grains dislodge, increasing the cutting force. Before new diamond abrasive grains appear, the bonding strength affects the cutting force when the bonding agent collides with the workpiece. To compare the diamond tool end faces after DGM and HRUM, a super depth-of-field video microscope (DVM6, Leica, Wetzlar, Germany) was used for preliminary observation. The results are shown in Figure 13a,b. It can be seen that the exposed abrasive grains are more evenly distributed on the end face with ultrasonic vibration compared to those without ultrasonic vibration. Diamond grinding causes more abrasive wear due to friction between the abrasive grains and the surface of the hard-working material, resulting in the blunting of the sharp corners of the abrasive grains. Consequently, the diamond abrasive grains form a smaller plane. However, in HRUM, the ultrasonic vibration effect can reduce the grinding force and friction coefficient. Figure 13c demonstrates that under the grinding of the tool, Al_2_O_3_ ceramic is removed in the form of chips, but it also tends to adhere to the end face of the tool.

## 5. Conclusions

This paper proposes the HRUM technology and develops the HRUM machine tool to address the problems associated with the conventional DGM microstructure of hard and brittle materials. Finally, a comparative experimental study is conducted between HRUM and DGM ceramic small holes. It mainly includes three parts:

(1) The hardware part of the machine tool mainly includes the spindle module, micro-motion system module, ultrasonic machining tank module, and DAQ system module.
(2) A LabView-based controlled machining control system is developed, which includes modules such as motion selection, initialization, coarse tool setting, constant force tool setting, control machining, and the coordinate display module.
(3) The experimental results show that compared with DGM, HRUM can effectively reduce axial cutting forces, reducing binder adhesion and suppressing slippage while also improving tool-cutting ability and extending tool life under the same machining parameters. At the initial machining stage, HRUM successfully reduces chipping and other defects. However, it is not as effective in later stages of hole machining, where chipping still occurs. Moreover, the size of grit used impacts cutting forces, tool wear, and the number of holes machined. Larger grit sizes result in fewer machined holes.

## Figures and Tables

**Table 1:** NI6361 DAQ card main technical parameters.

Analog Input	Analog Output
Channel number: 8 differentials/16 single-ended	Channel number: 2
ADC resolution ratio: 16 bit	DAC resolution ratio: 16 bit
Sampling rate: Maximum—2.00 MS/s single-channel, 1.0 MS/s multi-channel; Minimum—none; Timing precision—50 ppm/sampling rate; Timer resolution—10 ns	Maximum update rate: 2.00 MS/s Timing precision: 50 ppm/sampling rate Timer resolution: 10 ns
Input coupling: DC	Output coupling: DC
Input range: ±10 V, ±5 V, ±2 V; ±1 V; ±0.5 V; ±0.2 V and ±0.1 V	Output range: ±10 V, ±5 V and ±external reference

**Table 2:** Main material parameters of the microfine ultrasonic vibration system.

Component	Material	Density (kg/m^3^)	Longitudinal Wave Velocity-c (m/s)
Piezoceramic	PZT-8	7.6 × 10^3^	4720
Post matching block	45 steel	7.9 × 10^3^	5169
Front matching block	Titanium alloy	4.5 × 10^3^	5070

**Table 3:** Properties of Al_2_O_3_ ceramic.

**Main components**	99% Al_2_O_3_	
**Material properties**	Density	3.8 g/cm^3^
**Physical properties**	Hardness	HV 1700
	Flexural strength	3500 K_gf_/cm^2^
	Compressive strength	30,000 K_gf_/cm^2^
	Fracture toughness	4 Mpa m ^1/2^

**Table 4:** Machining parameters.

Group	Test	Grain Size (Mesh/mm)	Ultrasonic Power (%)	Feed Speed (mm/s)	Rotation Speed (r/min)
1	HRUM	80/0.18	60	0.01	10,000
2		160/0.11			
3		200/0.09			
4	DGM	80/0.18	0	0.01	10,000
5		160/0.11			
6		200/0.09			

## Data Availability

Data sharing does not apply to this article.
